# Natural Sources and Pharmacological Properties of Pinosylvin

**DOI:** 10.3390/plants11121541

**Published:** 2022-06-09

**Authors:** Saad Bakrim, Hamza Machate, Taoufiq Benali, Nargis Sahib, Imane Jaouadi, Nasreddine El Omari, Sara Aboulaghras, Sneh Punia Bangar, José Manuel Lorenzo, Gokhan Zengin, Domenico Montesano, Monica Gallo, Abdelhakim Bouyahya

**Affiliations:** 1 Molecular Engineering, Valorization and Environment Team, Polydisciplinary Faculty of Taroudant, Ibn Zohr University, Agadir B.P. 32/S, Morocco; s.bakrim@hotmail.com; 2 Laboratory of Biotechnology, Environment, Agri-Food and Health (LBEAS), Faculty of Sciences, University Sidi Mohamed Ben Abdellah (USMBA), Fez B.P. 1796, Morocco; hamza.mechchate@usmba.ac.ma; 3 Environment and Health Team, Polydisciplinary Faculty of Safi, Cadi Ayyad University, Sidi Bouzid B.P. 4162, Morocco; benali.taoufiq@gmail.com; 4 Laboratoire d’Amélioration des Productions Agricoles, Biotechnologie et Environnement (LAPABE), Faculté des Sciences, Mohammed Premier University, Oujda 60000, Morocco; n.sahib@ump.ac.ma; 5 Laboratory of Organic Chemistry, Catalysis and Environment, Department of Chemistry, Faculty of Sciences, Ibn Tofail University, B.P.:133, Kenitra 14000, Morocco; jaouadi.imane@gmail.com; 6 Laboratory of Histology, Embryology, and Cytogenetic, Faculty of Medicine and Pharmacy, Mohammed V University in Rabat, Rabat 10100, Morocco; nasrelomari@gmail.com; 7 Physiology and Physiopathology Team, Faculty of Sciences, Genomic of Human Pathologies Research, Mohammed V University in Rabat, Rabat 10100, Morocco; sara.aboulghras@gmail.com; 8 Department of Food, Nutrition and Packaging Sciences, Clemson University, Clemson, SC 29634, USA; snehpunia69@gmail.com; 9 Centro Tecnológico de la Carne de Galicia, Rúa Galicia Nº 4, Parque Tecnológico de Galicia, San Cibrao das Viñas, 32900 Ourense, Spain; jmlorenzo@ceteca.net; 10 Facultade de Ciencias, Universidade de Vigo, Área de Tecnoloxía dos Alimentos, 32004 Ourense, Spain; 11 Department of Biology, Science Faculty, Selcuk University, Konya 42130, Turkey; gokhanzengin@selcuk.edu.tr; 12 Department of Pharmacy, University of Naples Federico II, Via D. Montesano 49, 80131 Naples, Italy; domenico.montesano@unina.it; 13 Department of Molecular Medicine and Medical Biotechnology, University of Naples Federico II, Via Pansini, 5, 80131 Naples, Italy; 14 Laboratory of Human Pathologies Biology, Department of Biology, Faculty of Sciences, Mohammed V University in Rabat, Rabat 10100, Morocco

**Keywords:** pinosylvin, pharmacological property, signaling pathway, antimicrobial, cancer

## Abstract

Pinosylvin (3,5-dihydroxy-*trans*-stilbene), a natural pre-infectious stilbenoid toxin, is a terpenoid polyphenol compound principally found in the Vitaceae family in the heartwood of *Pinus* spp. (e.g., *Pinus sylvestris*) and in pine leaf (*Pinus densiflora*). It provides defense mechanisms against pathogens and insects for many plants. Stilbenoids are mostly found in berries and fruits but can also be found in other types of plants, such as mosses and ferns. This review outlined prior research on pinosylvin, including its sources, the technologies used for its extraction, purification, identification, and characterization, its biological and pharmacological properties, and its toxicity. The collected data on pinosylvin was managed using different scientific research databases such as PubMed, SciFinder, SpringerLink, ScienceDirect, Wiley Online, Google Scholar, Web of Science, and Scopus. In this study, the findings focused on pinosylvin to understand its pharmacological and biological activities as well as its chemical characterization to explore its potential therapeutic approaches for the development of novel drugs. This analysis demonstrated that pinosylvin has beneficial effects for various therapeutic purposes such as antifungal, antibacterial, anticancer, anti-inflammatory, antioxidant, neuroprotective, anti-allergic, and other biological functions. It has shown numerous and diverse actions through its ability to block, interfere, and/or stimulate the major cellular targets responsible for several disorders.

## 1. Introduction

Pinosylvin (3,5-dihydroxy-*trans*-stilbene), a natural pre-infectious stilbenoid toxin, is a stilbenoid polyphenol component mostly contained in the Pinaceae family, particularly in the heartwood of *Pinus* spp. (e.g., *Pinus sylvestris*) and in pine leaf (*Pinus densiflora*). Traditionally, different parts of pine trees have been used in East Asia for various purposes, such as treating liver toxicity, gastric disorders, and inflammation. In South Korea, pine needles were commonly consumed as tea and food [1]. The most stilbenes are contained in the Vitaceae family of plants containing, represented by the famous wine grape *Vitis vinifera* L., which is among the most abundant sources of new stilbenes currently known, along with other families, such as Fabaceae, Dipterocarpaceae, and Gnetaceae [2]. Pinosylvin is suggested as a functional compound responsible as a defense mechanism against pathogens and insects for a wide range of plants, especially pines [3]. They are also found in most berries or fruits, but can also be found in other types of plants, such as mosses and ferns [4]. Pinosylvin generates phytoalexins via a reaction between malonyl-CoA and cinnamoyl-CoA under the influence of different biotic and abiotic stresses like wounds, herbivores, fungi, ozone, and ultraviolet light [5]. Pinosylvin is widely explored for its relevance to plants and its characteristics favorable to human health, as it possesses several biological properties, including antimicrobial, anti-inflammatory, anticancer, antioxidant, neuroprotective, and antiallergic characteristics. Pharmacological examination of pinosylvin derivatives has shown a broad range of biological effects. In fact, they have antibacterial activity against different human pathogenic bacteria (Gram-positive and Gram-negative) such as *Staphylococcus aureus*, *Escherichia coli*, *Listeria monocytogenes*, *Lactobacillus plantarum*, *Salmonella infantis*, *Pseudomonas fluorescens*, *Campylobacter jejuni*, and *Campylobacter coli* [6,7,8]. Furthermore, pinosylvin exhibited significant antifungal effects against pathogenic fungi such as *Candida albicans*, *Saccharomyces cerevisiae*, *Trametes versicolor*, *Phanerochaete chrysosporium*, *Neolentinus lepideus*, *Gloeophyllum trabeum*, *Postia placenta*, *Rhizoctonia solani*, *Sclerotinia homoeocarpa*, etc. [9,10]. Also, pinosylvin, as a natural molecule, has been widely investigated in vitro and in vivo for its excellent anti-inflammatory potential, as evidenced in several studies [11,12,13,14,15]. The antioxidant activity of pinosylvin has been extensively studied by different investigators, not only in isolated case studies but also in association with several diseases such as rheumatoid arthritis, age-related diseases (age-related macular degeneration (AMD) and Alzheimer’s disease), and oligoasthenospermia by reducing oxidative stress via the nuclear factor erythroid 2-related factor 2 (Nrf2)/antioxidant response element (ARE) pathway [16,17,18,19]. Pinosylvin is well recognized for its potential chemopreventive activity against cancer [20,21] even at low concentrations [22]. It showed anti-cancer activity against nasopharyngeal cancer [20], prostate cancer [23], fibrosarcoma [5], colorectal cancer [21], and oral cancer [20]. Indeed, in HCT116 colorectal cancer cells, pinosylvin was found to block the activation of proteins that play a role in the FAK/c-Src/ERK and PI3K/Akt/GSK-3b signaling pathways [21]. Additionally, in cultured HT1080 human fibrosarcoma cells, pinosylvin inhibited the production of matrix metalloproteinase (MMP)-2, MMP-9, and membrane type 1-MMP. The antimetastatic action of pinosylvin was associated with the downregulation of MMP-9 and cyclooxygenase-2 (COX-2) [5]. Despite the availability of certain investigations that have highlighted the different pharmacological functions of pinosylvin and its derivatives, to the best of our knowledge, no critical review has been carried out to provide suggestions for potential future clinical uses of this bioactive molecule. This synthesis article aims to provide a comprehensive review of the characteristics of this secondary metabolite, namely its sources, extraction technologies, purification, identification, characterization, and pharmacological and biological properties. We hope that this review will give a novel background for further investigations on this deterrent secondary metabolite and its pharmacological actions in order to explore new pharmaceutical opportunities for this natural molecule.

## 2. Sources of Pinosylvin

Pinosylvin, known as 3,5-dihydroxy-trans-stilbene, was first isolated by Erdtman in 1939 from extracts derived from *Pinus sylvestris*, hence the name pinosylvin [24]. It is a natural stilbenoid belonging to the phenolic group of compounds. Pinosylvin is found in a wide range of plant species, particularly in the leaves and wood of various *Pinus* species (Table 1).

## 3. Technology of Extraction and Purification

Stilbenes have attracted increasing interest in recent years for their health benefits in preventing disease. For these reasons, their extraction and subsequent purification are particularly interesting for the production of high-quality extracts. Due to its very low occurrence in plants, and the environmental and chemical hazards associated with multi-step isolation and purification processes, the extraction of pinosylvin from plants was difficult and unsustainable. These drawbacks have led scientists to introduce alternative production sources into the stilbene isolation process, such as the callus culture, plant culture, cell suspension culture, hairy root culture, genetically-modified plants, and the introduction of the stilbene biosynthetic genes into microbial hosts.

These different biotechnological approaches have been applied to avoid the formation of many undesirable biproducts of high medicinal risk in the extracts obtained by chemical synthesis. These biproducts subsequently necessitate a more complicated purification process, such as sequential flash chromatography in two repetitions in gradient mode during the mobile phase with cyclohexane (CX) and ethyl acetate (EtOAc) [50].

### 3.1. Pinosylvin Production in Callus Cultures and Cell Suspension Cultures

In 1961, Jorgensen showed that mechanical damage applied to the bark and cambium of red pine causes fungal penetration of the sapwood into the stems and roots. This fungal penetration affects the dying tissues and induces the formation of pinosylvin and its monomethyl ether, which are absent in the healthy sapwood [51]. At temperatures where cellular activity is possible, pinosylvin is formed by living cells in response to desiccation, causing slow tissue death in sections of living branches or callus tissues, thus producing the stilbenes.

In a study reported by Koo et al. [40], the authors have established a system of production of pinosylvin stilbene and its derivatives using the in vitro culture of *Pinus strobus* L. callus. From a culture of mature zygotic embryos in 1/2 Litvay medium with 1.0 mg/L 2.4-D and 0.5 mg/L BA, calli were obtained and the accumulation of pinosylvin significantly increased in prolonged callus cultures. In 1984, and for the first time, Schöppner and Kindl [49] described the purification of pinosylvin synthase from cell suspension cultures of peanuts using column chromatography. This study showed that from hypocotyls of 4-day-old seedlings, peanut cell cultures were initiated and propagated as callus cultures.

In a study reported by Lange et al. [52], the accumulation of the stilbenes pinosylvin and pinosylvin 3-O-methyl ether in methanolic cell extracts was induced by a cell suspension of treatment of *Pinus sylvestris* L. cultures with an elicitor preparation of the pine needle pathogen.

### 3.2. Microbial Biosynthesis of Pinosylvin

The bioproduction of pinosylvin was achieved by genetic engineering of the host strain to integrate the heterologous pathways of plants with the host strain. *E. coli* was the commonly used host for pinosylvin production. The bacterial hosts have a short life cycle, high growth rate, and easy genetic manipulation, and, therefore, significant overexpression of proteins and enzymes. At the same time, they severely lack the expression of large proteins and the post-translational modifications necessary for the correct folding and functional activity of recombinant proteins [53,54]. Therefore, pathway engineering is one of the pioneering methods in *E. coli* design (50% bioconversion rate) which facilitated the bioproduction of stilbenes in different micro-organisms to produce value-added bioactive pinosylvin [55].

Interestingly, another study described the development of an *E. coli* platform strain to produce the stilbene pinosylvin found in the heartwood of pines [56]. The authors of this study reported low pinosylvin concentrations (3 mg/L) after the optimization of gene expression and evaluation of different construction environments. To promote the production of pinosylvin stilbene, the authors added cerulenin to increase the intracellular reserves of malonlyl-CoA and subsequently obtained higher concentrations of pinosylvin of up to 70 mg/L from glycose and 91 mg/L by adding L-phenylalanine. Similar results were obtained in a study conducted by Xu et al. [57], who evaluated the biosynthetic pathway for pinosylvin production in engineered *E. coli.* It was shown in this study that the excessive accumulation of the precursor malonyl-CoA leading to malonylation of the biosynthetic enzymes decreases pinosylvin yield. In order to mitigate this decrease, several metabolic engineering techniques (PTM, PTM-ME) have been established to maintain an optimal level of intracellular acyl-CoA concentration, and thus increase the pinosylvin yield. Liang et al. [58] investigated an alternative approach to pinosylvin production using three bioengineering strategies to develop a simple and economical process for pinosylvin biosynthesis in *E. coli*. The authors were able to produce 47.49 mg/L of pinosylvin from glycerol, using these combinatory processes by promoting the expression of the pinosylvin pathway enzymes, increasing the level of the key precursor of pinosylvin bioproduction (malonyl-CoA) in the *E. coli* cell. The final step was to introduce phenylalanine super-producing *E. coli* to produce trans-cinnamic acid which is a precursor of pinosylvin. Other researchers have also established metabolic engineering techniques for *E. coli* for the biosynthesis of stilbene pinosylvin [59,60,61].

## 4. Technology of Identification and Characterization

Chromatographic analyses (GC-MS, LC-MS, GC-FID or HPLC) are frequently used to identify and characterize stilbenes using stilbene standards. In 1999, Holmgren et al. [62] used diffuse reflectance Fourier transform infrared spectroscopy (DRIFT) and FT-Raman near-infrared spectroscopy (NIR) to detect the presence of pinosylvin and its derivatives in the wood of *Pinus sylvestris* L. by a simple visual inspection of uniform wood blocks in disc form. Roupe et al. [63] developed a simple and novel high-performance liquid chromatography (HPLC) method to simultaneously determine pinosylvin and its metabolic products in rat serum and liver microsomes. The method consists of a preliminary precipitation of serum or microsomes with acetonitrile after adding an internal standard. The separation was then performed on a tris-3,5 dimethyl phenyl carbamate amylose column with UV detection at 308 nm. In another study performed by Ekeberg et al. [64], the quantitative identification of *P. sylvestris* L. heartwood extracts, including pinosylvin and its derivatives, was carried out using gas chromatography (GC) with flame ionization detection (FID). Similarly, a study on Scots pine/spruce wood residues from Norway conducted by Poljanšek et al. [50], describes the qualitative and quantitative analysis of the obtained extracts in terms of pinosylvin and pinosylvin monomethyl ether performed uisng gas chromatography with a flame ionization detector (GC-FID) and gas chromatography with mass spectrometry (GC-MS).

Somewhat removed from plant samples, Preusz et al. [65] conducted a study on organic residues in the form of black stains found at the sites of the ancient ports of Pyrgi and Castrum Novum on the Tyrrhenian coast, in which pinosylvin monomethyl ether was identified and confirmed for the first time in archaeological samples using GC-MS and HPLC with fluorimetric detection.

## 5. Biological and Pharmacological Properties

**Figure 1:**
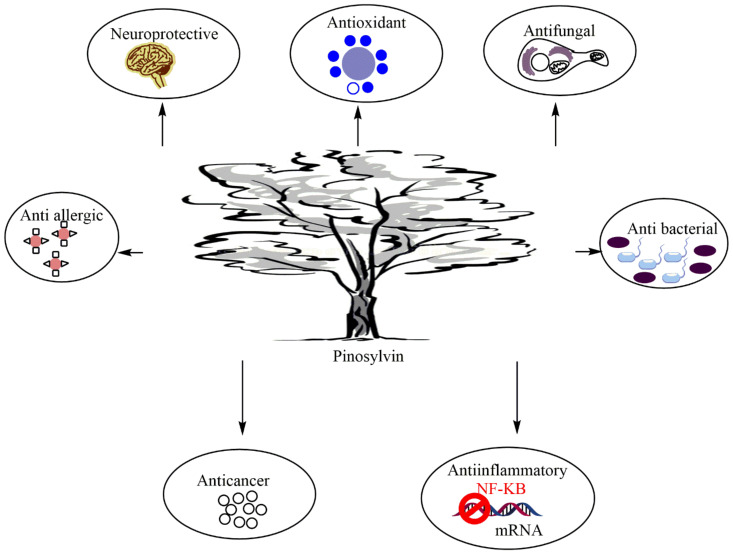
Major pharmacological properties of pinosylvin.

As evidenced in several investigations, pinosylvin was found to exhibit a wide range of biological and pharmacological properties, including antimicrobial [66], anti-inflammatory [11], antioxidant [17], anticancer [5,23], neuroprotective [67], and anti-allergic [45] effects (Figure 1).

### 5.1. Antimicrobial Activity

With the increasing problems of the persistence and emergence of microbial resistance, much attention was given to the identification of new antimicrobial drugs derived from natural bioactive compounds [68,69,70,71]. Pinosylvin has been widely investigated for its health benefits and biological activities, including its antimicrobial effects. Lee et al. [66] showed the role of pinosylvin as an antimicrobial agent against various human pathogens, including Gram-positive (*S. aureus*) and Gram-negative (*E. coli*) bacteria, fungi (*C. albicans*), and yeasts (*S. cerevisiae*). *C. albicans* and *S. cerevisiae* appeared to be more sensitive to pinosylvin with minimal inhibitory concentration (MIC) values of 62.5 and 125 µg/mL, respectively, while the MIC for *E. coli* and *S. aureus* was 250 µg/mL. Moreover, pinosylvin extracted from the knot wood and bark of different *Pinus* species exhibits potent antimicrobial activity, effectively inhibiting the growth of a broad spectrum of pathogenic strains, including *Bacillus cereus*, *S. aureus*, *L. monocytogenes*, *L. plantarum*, *E. coli*, *S. infantis*, *P. fluorescens*, *C. albicans*, *S. cerevisiae*, *Aspergillus fumigatus*, and *Penicillium brevicompactum*, with inhibition diameters ranging from 19 ± 1 to 101 ± 6 mm. Sousa and collaborators [8] evaluated the potential interaction between pinosylvin and four antibiotics (tetracycline, chloramphenicol, erythromycin, and ciprofloxacin) against *Arcobacter butzleri* using checkerboard titration assays. Based on FICI values, no synergistic effects were observed for pinosylvin/four antibiotic combinations, while pinosylvin showed additive interactions on all the tested antibiotics, except ciprofloxacin. In addition, these researchers investigated the ability of pinosylvin to modulate the efflux pump activity using ethidium bromide (EtBr) accumulation assays. The results showed that pinosylvin causes a higher intracellular accumulation of EtBr, elucidating that it may attenuate the activity of efflux pumps (EPs) [8]. Overall, these findings shed light on the use of pinosylvin as a resistance modulator to control the decreasing susceptibility of *A. butzleri* to antibiotics and suggest the potential of pinosylvin as an efflux pump inhibitor. Furthermore, prenylation of stilbenes, including pinosylvin has been shown to enhance their antibacterial activity, which is explained by MIC values. In this regard, Bruijn et al. [72] demonstrated that prenylated pinosylvin derivatives isolated from *Rhizopus* extract exhibit potent antimicrobial activity against methicillin-resistant *Staphylococcus aureus* (MRSA), especially chiricanine A with a MIC value of 12.5 µg/mL.

As a natural compound, pinosylvin has potential applications in the development of antimicrobial food packaging systems due to its inherent antimicrobial activity, especially against Campylobacter spp. [6,7]. Indeed, it has been shown that pinosylvin or its inclusion complexes (ICs) with modified cyclodextrins (hydroxypropyl-b-cyclodextrin and hydroxypropyl-g-cyclodextrin) were able to inhibit the growth of *Campylobacter jejuni* and *Campylobacter coli* American type culture collection (ATTC) reference strains and clinical isolates [73]. The MIC values were between 25 to 50 mg/mL for the pure compound and between 16 and 64 mg/mL for the ICs. Furthermore, time-kill assays showed that pinosylvin ICs exhibit bactericidal action on both *Campylobacter* species at 37 °C and even at 4 or 20 °C [73]. Flow cytometric analysis shows that the mechanism behind this bactericidal action may mainly involve membrane damage mediated by the impairment of various cellular functions such as membrane polarization, permeability, and efflux activity [73]. These promising data make these pinosylvin ICs valuable lead compounds used in active food packaging to eradicate *Campylobacter* spp. in fresh poultry products. In this context, in their recent findings, the same authors demonstrated the role of coated pads containing pinosylvin ICs in controlling fresh chicken meat from *Campylobacter* contamination [7]. The above-mentioned compound exhibited effective in vitro bactericidal activity against *C. jejuni* with more than 99% colony count inhibition, even at the lowest concentrations (0.08 mg/cm^2^). In vivo tests on chicken exudates and chicken fillets have also shown that these active pads exhibit promising anti-Campylobacter activity at 37 °C and 4 °C [7]. Additionally, coated pads-pinosylvin ICs are also effective against other major chicken foodborne bacteria, suggesting future uses of this coating as a new alternative to control the microbial growth in packaged chicken meat [7].

On the other hand, the antifungal potential of pinosylvin and pinosylvin monomethyl ether isolated from pine knot extract was assessed in vitro against *Plasmopara viticola*. This study showed that pinosylvin exhibits promising antimildew properties, inducing significant inhibition of zoospore mobility (IC_50_ = 34 μM) and mildew development (IC_50_ = 23 μM) [43]. These findings are corroborated by those described by other authors. Indeed, pinosylvin and pinosylvin monomethyl ether from *Pinus* trees have already demonstrated significant antifungal effects against white rot (*Trametes versicolor* and *Phanerochaete chrysosporium*) and brown-rot (*Neolentinus lepideus*, *Gloeophyllum trabeum*, and *Postia placenta*) [9] fungi. Furthermore, pinosylvin from the methylene chloride fraction of *Pinus densiflora* showed effective antifungal activity against plant pathogens such as *Rhizoctonia solani* AG1-1B, *R. solani* AG2-2IV, *R. cerealis*, and *S. homoeocarpa*. *S. homoeocarpa* showed the highest sensitivity with the lowest mean EC_50_ value (8.426 μg/mL), whereas among the *Rhizoctonia* pathogens, *R. cerealis* had the highest mean EC_50_ value (99.832 μg/mL). Pinosylvin could be a valuable lead compound for developing new effective and ecofriendly antifungal agents [10].

### 5.2. Anti-Inflammatory Activity

Over the past decades, several scientists have dedicated their efforts to developing novel anti-inflammatory drugs from natural molecules to overcome the serious and excessive side effects of current drugs [74,75]. As a natural molecule, pinosylvin has been extensively investigated (in vitro and in vivo) for its excellent potential anti-inflammatory effects (Table 2).

**Figure 2:**
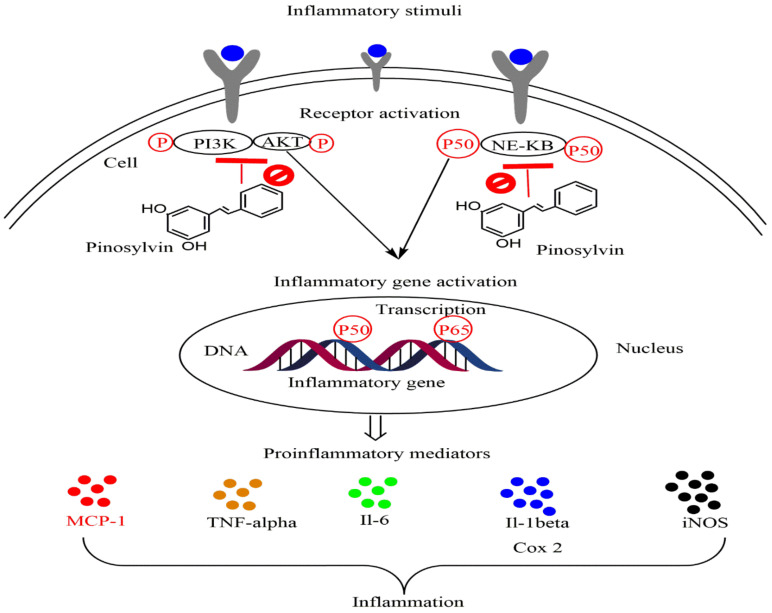
Anti-inflammatory effects of pinosylvin. This figure illustrates the ability of pinosylvin to reduce the expression of some proinflammatory cytokines and enzymes, probably via the inactivation of NF-κB and the PI3K/Akt pathway. Abbreviations: NF-κB, nuclear factor kappa B; NO, nitric oxide; COX-2, cyclooxygenase-2; iNOS, inducible nitric oxide synthase; IL6, interleukin 6; MCP1, monocyte chemotactic protein 1; TNF-α, tumor necrosis factor-α.

Research results by Park and colleagues (2005) [84] showed that pinosylvin downregulates the production of proinflammatory mediators such as prostaglandin E_2_ (PGE_2_) and nitric oxide (NO) in a dose-dependent manner. This effect was directly related to COX and inducible nitric oxide synthase (iNOS) inhibition. Moreover, pinosylvin significantly inhibited other key inflammatory enzymes, interleukin 6 (IL6) (IC_50_ = 32.1 µM) and monocyte chemotactic protein 1 (MCP1) (IC_50_ = 38.7 µM) [11]. Additionally, the in vivo investigation measuring carrageenan-induced paw edema in male C57BL/6 mice showed that pinosylvin at a dose of 30 mg/kg significantly reduced the inflammatory response by downregulating the production of inflammatory cytokines IL6, MCP1, and NO compared to an LY294002-treated group [11]. The similar anti-inflammatory effects of pinosylvin to those of the known commercial phosphatidylinositol-3 kinase (PI3K) inhibitor LY294002 suggest that these effects may be mediated by the inhibition of the PI3K/Akt pathway (Figure 2).

Furthermore, treatment with pinosylvin was shown to significantly inhibit stimulation-induced NO production of murine macrophages with lipopolysaccharide (LPS) in a dose-dependent manner, with an IC_50_ value of 39.9 μM compared to reference _L_-NMMA (IC_50_ = 30.7 μM) [79]. In addition, pinosylvin suppressed iNOS gene expression via downregulation of interferon regulatory factor 3 (IRF-3) and interferon-E (IFN-E) expression related to TIR-domain-containing adapter-inducing interferon-β (TRIF) mediated signaling pathway. These events were then associated with the suppression of JAK kinase phosphorylation, which decreased the phosphorylation of signal transducer and activator of transcription-1, one of the iNOS transcriptional activators [79].

Since the substitution patterns of the trans-stilbene have been shown to enhance various biological properties, Park et al. [78] assessed the substitutions of the dihydroxy group in pinosylvin with different lipophilic derivatives on LPS-induced RAW 264.7 cells. The results showed that the synthesized pinosylvin derivatives, especially 3,5-dimethoxy-trans-stilbene and 3-hydroxy-5-benzyloxy-trans-stilbene, significantly suppress COX-2 mRNA expression-mediated PGE_2_ production. On the other hand, pinosylvin treatment at doses of 5 and 10 µM greatly enhanced human RPE cells from oxidative stress. The expression levels of heme oxygenase-1 (HO-1), an enzyme with anti-inflammatory and immunomodulatory activities, were upregulated by pinosylvin treatment and markedly correlated with cell survival [70]. These findings demonstrated the role of pinosylvin treatment in the protection against oxidative stress, induction of HO-1 expression in human RPE cells and, therefore, potential health promotion against oxidative stress and aging-related diseases such as AMD and Alzheimer’s disease [77].

Interestingly, in addition to its ability to reduce the concentration of reactive oxygen and nitrogen species, pinosylvin has been shown to potentiate the therapeutic efficacy of methotrexate (MTX), an immunosuppressive drug in arthritis treatment. Indeed, Bauerova et al. [16] showed that the treatment of AA in rats with pinosylvin in combination with MTX (Orale doses of 50 mg/kg b.w. for PIN and 0.4 mg/kg b.w. for MTX) significantly reduced oxidative stress via upregulation of HO-1 expression in the lungs and reduction in plasmatic thiobarbituric acid reactive substances (TBARS) as well as markedly decreased lipoxygenase (LOX) activity in the lungs.

Ankyrin subtype 1 protein (TRPA1) has been involved in various inflammatory responses. Its suppression may provide promising targets for the treatment of many pathological conditions related to acute pain, inflammation, and hyperalgesia. In this respect, Moilanen and colleagues (2018) conducted their research to investigate the effect of pinosylvin on TRPA1 in vitro by measuring transient receptor potential ankyrin subtype 1 protein (TRPA1)-mediated Ca^2+^ influx and membrane currents. The findings reported a dose-dependent inhibitory effect of pinosylvin (IC_50_ = 16.7 μM) on AITC-induced TRPA1-mediated responses. In vivo experiments using AITC-induced paw inflammation as a model demonstrated that pinosylvin treatment effectively reduced the formation of paw edema, attenuating the production of inflammatory cytokine IL-6 at the site of the inflammation [15].

### 5.3. Antioxidant Activity

The antioxidant activity of pinosylvin was extensively studied by different researchers, not only as an isolated study case (ORAC, ABTS^+^, and FRAP in vitro assays), but in relation to many diseases such as rheumatoid arthritis, age-related diseases, and oligoasthenospermia [16,17,18,19] (Table 3).

Bauerova et al. [16] assessed the impact of the treatment on selected parameters in AA (Alko, alcohol) rats when administered pinosylvin for 28 days as a monotherapy and in combination with methotrexate (MTX). The experiment included healthy controls, untreated AA, and AA given 50 mg/kg b.w. pinosylvin daily p.o. AA was monitored using hind paw volume, C-reactive protein, MCP-1 activity, TBARS, F_2_-isoprostanes in plasma, g-glutamyltransferase activity in the spleen, lung LOX activity, HO-1 activity, and nuclear factor kappa B (NF-κB). Pinosylvin monotherapy enhanced NF-κB activation in the liver and lung, HO-1 expression and LOX activity in the lung, plasma MCP-1 levels (on the 14th day), and plasmatic levels of F_2_-isoprostanes. The reduction in OS (an increase in HO-1 expression in the lungs and a reduction in plasmatic TBARS) and decrease in LOX activity in the lungs were substantial contributions of pinosylvin.

The pathophysiology of rheumatoid arthritis is strongly influenced by oxygen metabolism. Patients with rheumatoid arthritis have an altered antioxidant defense capacity barrier, which links oxidative stress, inflammation, and the immune system. Drafi et al. [17] investigated the impact of pinosylvin in monotherapy for the treatment of AA. Indeed, pinosylvin (30 mg/kg body mass daily per os) was provided in monotherapy to rats with AA for 28 days. In rats, parameters such as changes in hind paw volume and arthritis score were measured as indicators of destructive arthritis-related clinical changes, with determination of oxidative indicators, plasmatic levels of TBARS, and the latency of Fe^2+^-induced lipid peroxidation (tau-FeLP) in plasma and the brain. CRP levels in the blood and glutamyltransferase (GGT) activity in the spleen and joints have been used as inflammatory indicators. Pinosylvin failed to significantly reduce the arthritic score in arthritic animals compared to untreated arthritic animals. Administration of pinosylvin somewhat reduced GGT activity in the spleen. Pinosylvin was less effective in reducing oxidative damage as determined by plasma TBARS levels.

Jančinová et al. [81] conducted their investigation to evaluate the effects of natural stilbenoid pinosylvin on neutrophil activity in vitro and experimental arthritis and to determine whether protein kinase C (PKC) activation functioned as an assumed target of pinosylvin action. The oxidative burst was assessed using enhanced chemiluminescence from neutrophils from fresh human blood. Flow cytometry was used to analyze neutrophil viability, and Western blotting was used to determine PKC phosphorylation. Adjuvant arthritis was produced in Lewis rats using heat-killed Mycobacterium butyricum, and the animals received pinosylvin (30 mg/kg, p.o.) daily for 21 days after arthritis was established. Pinosylvin (10 and 100 µmol/L) greatly reduced the generation of extracellular and intracellular oxidants and efficiently inhibited PKC activation triggered by phorbol myristate acetate (0.05 µmol/L) in isolated human neutrophils. However, inhibition did not occur due to neutrophil damage or increased apoptosis. Blood neutrophil counts were considerably elevated in arthritic rats, as was whole blood chemiluminescence (spontaneous and PMA-stimulated). The injection of pinosylvin reduced the number of neutrophils and considerably lowered the number of reactive oxygen species in blood. Pinosylvin is a potent inhibitor of neutrophil activity and has the potential to be beneficial as an adjunctive drug in conditions related to chronic inflammation. The observed results qualified pinosylvin as an efficient inhibitor of neutrophil activity, suggesting that it could be beneficial as a supplemental therapy in pathological situations related to chronic inflammation.

Koskela et al. [77] studied the capacity of pinosylvin to control oxidative stress in human RPE cells. ARPE-19 cells were treated with pinosylvin (5 µM) for 6 h, and mRNA was extracted at four timepoints (2 h, 6 h, 12 h, and 24 h) to determine changes in the expression of Nrf2, sequestosome 1 (p62/SQSTM1), HO-1, and glutathione S-transferase pi 1 (GSTP1). To further understand the molecular mechanism underlying pinosylvin-mediated protection, ARPE-19 cells were transfected with p62 and Nrf2 siRNAs for 24 h, and the roles of p62, Nrf2, and its target gene HO-1 in protection against oxidative stress were investigated using quantitative real-time PCR (qRT-PCR) and cell viability assay. At doses of 5 and 10 µM, pinosylvin dramatically improved cell survival against oxidative stress and increased the expression of HO-1, an enzyme with antioxidant, anti-inflammatory, and immunomodulatory capabilities, and was substantially linked to cell survival. However, pinosylvin treatment did not influence the expression of Nrf2 or its target genes, p62 or GSTP1, while having a strong effect on the expression of HO-1, another Nrf2-controlled gene. RNA interference study verified the importance of Nrf2 and HO-1 in PS-mediated protection against oxidative stress, whereas the contribution of p62 seemed minor at the levels of gene expression and cell viability. According to the findings of this research, pinosylvin therapy protects against oxidative stress by inducing HO-1 in human RPE cells.

In the study by Mačičková et al. [80], the research focused on the impact of pinosylvin on the development of adjuvant arthritis in rats. AA was developed in male Lewis rats using a single intradermal injection of *Mycobacterium butyricum* in inadequate Freund’s adjuvant. Pinosylvin (30 mg/kg) was regularly given orally to arthritic animals. The therapy consisted of administering the chemicals examined from day 0 (day of immunization) to day 28 (experimental day), measuring several parameters, namely change in hind paw volume (HPV) at days 14, 21, and 28, joint chemiluminescence (CL), and myeloperoxidase (MPO) activity in hind paw joint homogenates (day 28). Arthritic animals treated with pinosylvin substantially reduced HPV at days 14 and 28. In contrast to untreated mice, pinosylvin lowered joint CL and joint homogenate MPO activity. This molecule demonstrated a favorable anti-inflammatory and antioxidant impact on oxidative stress-induced biochemical alterations in AA according to the three functional measures.

Rodríguez-Bonilla et al. [18] measured the antioxidant capacity of pinosylvin using a variety of analytical methodologies (ORAC, ABTS^+^, or FRAP). Pinosylvin showed high antioxidant and free radical scavenging activity in all experiments due to phenolic hydroxy groups.

Considering that chronic oxidative stress eventually leads to protein aggregation in combination with impaired autophagy, as seen in AMD, Tamminen et al. [85] investigated the effects of commercial natural pinosylvin extract, Retinari™, on electroretinogram, optical coherence tomogram, autophagic activity, antioxidant capacity, and inflammation markers in their study. For 10 weeks before the experiments, wild-type and NFE2L2 knockout mice were given either ordinary or Retinari™ chow. Retinari™ therapy restored many retinal functions, with a- and b-wave amplitudes in electroretinogram responses being considerably preserved. Furthermore, this treatment reduced retinal thinning in NFE2L2 mutant animals that showed lower ubiquitin-tagged protein accumulation and local overexpression of complement factor H and the antioxidant enzymes superoxide dismutase 1 and catalase. Accordingly, in the NFE2L2 KO illness model, the therapy decreased chronic oxidative stress while maintaining retinal function and shape. The findings suggest that taking pinosylvin supplements may reduce the likelihood of developing age-related macular degeneration and halt its development.

Pinosylvin, a resveratrol analogue developed by Wang et al. [19], has been thoroughly studied in the treatment of oligoasthenospermia. They explored the molecular basis for improved sperm parameters in a mouse model of oligoasthenospermia produced using busulfan (BUS) therapy at 6 mg/kg b.w. Mice were given varying concentrations of pinosylvin daily for two weeks after receiving busulfan treatment. Then, epididymal sperm concentration and motility were evaluated and testicular histology was performed. Levels of serum hormones, including testosterone (T), luteinizing hormone (LH), and follicle-stimulating hormone (FSH), were tested using ELISA kits designed for each hormone. RNA sequencing was used to establish testicular mRNA expression profiles. Quantitative real-time PCR, Western blotting, and ELISA were used to confirm these results. After BUS therapy, pinosylvin improved epididymal sperm concentration and motility, increased testosterone levels, and facilitated morphological testicular recovery. The antioxidant glutathione peroxidase 3 dramatically decreased oxidative stress through the Nrf2/ARE-dependent antioxidant. Pinosylvin improved oligoasthenospermia in this mouse model by reducing oxidative stress via the Nrf2-ARE pathway.

### 5.4. Anticancer Activity

Pinosylvin is a functional compound in *Pinus* species known to exhibit potential cancer chemopreventive activity [20,21], even at low concentrations [22]. Based on that, the main concern of the researchers was to reveal its underlying molecular mechanisms [5] as well as its potential against resistant types of cancer [23] and metastasis [45]. (Table 4).

**Figure 3:**
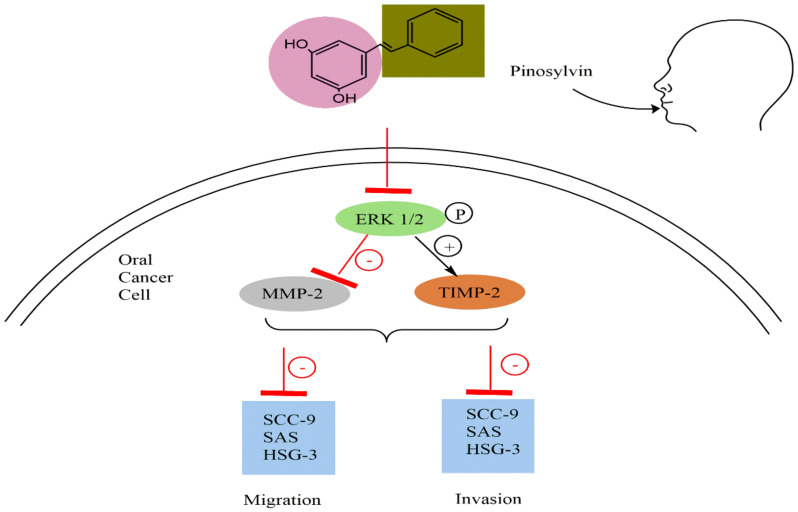
Anticancer activity of pinosylvin against oral cancer cells. Pinosylvin suppressed the invasion and migration of oral cancer cells by inhibiting the phosphorylation of ERK1/2 protein expression in SAS and SCC-9 cells. Abbreviations: MMP-2, matrix metalloproteinase-2; TIMP-2, tissue inhibitor of metalloproteinase-2; ERK, extracellular signal-regulated kinase.

The effects of pinosylvin on the migration and invasion of human oral cancer cells remain unknown, as do the underlying processes. Chen et al. [20] evaluated the effects of varying concentrations of pinosylvin (0–80 μM) on the metastatic and invasive capacities of SAS, SCC-9, and HSC-3 cells. Pinosylvin suppressed matrix metalloproteinase-2 (MMP-2) enzyme activity and lowered its protein level in Western blotting and gelatin zymography assays but enhanced the expression of tissue inhibitors of metalloproteinase-2 (TIMP-2). Pinosylvin also inhibited the migration of oral cancer cells (SAS, SCC-9, and HSC-3) in the wound healing experiment and using the transwell technique. Furthermore, this substance inhibited the phosphorylation of ERK1/2 protein expression in SAS and SCC-9 cells (Figure 3).

These findings suggest that pinosylvin may be a promising anticancer drug to prevent oral cancer spread. Chuang et al. [90] aimed to examine the functional role of pinosylvin in nasopharyngeal carcinoma (NPC) cells (NPC039, NPCBM, and RPMI 2650). According to gap-closure and transwell assays, pinosylvin reduced the migration and invasion of NPC039 and NPCBM cells at increasing doses. It not only inhibited the activity of MMP2 enzymes, but also reduced the expression levels of MMP2 and MMP9 proteins. Pinosylvin inhibited the expression of vimentin and N-cadherin while dramatically increasing that of zonula occludens-1 and E-cadherin in NPC cells. It also inhibited the invasion and migration of NPC039 and NPCBM cells by modulating the p38, ERK1/2, and JNK1/2 pathways. According to the findings of this investigation, pinosylvin suppressed the migration and invasion of NPC cells.

There are currently few therapeutic options for castration-resistant prostate cancer (CRPC). A high-throughput screen of 4910 drugs and drug-like molecules was used in a study conducted by Ketola et al. [23] to detect antiproliferative substances on prostate cancer after androgen ablation therapy. The effects of compounds on cell survival were examined in androgen-ablated LNCaP prostate cancer cells, LNCaP cells cultured in androgens, and two non-malignant prostate epithelial cells (RWPE-1 and EP156T). Pinosylvin methyl ether (PSME) was a strong inhibitor of androgen-ablated LNCaP cell growth in cancer-specific antiproliferative drug validation assays. A genome-wide gene expression study in PSME-exposed cells was undertaken to obtain insight into growth inhibitory mechanisms in CRPC. In androgen-depleted LNCaP cells, pinosylvin affected the expression of genes involved in cell cycle, steroid, and cholesterol production. Reduced androgen-receptor expression and prostate-specific antigen in PSME exposed cells verified the decrease in androgen signaling. Taken together, our comprehensive screen revealed PSME as a new antiproliferative agent for CRPC. These findings provide a solid foundation for future preclinical and clinical investigations on CRPC treatment.

The capacity of pinosylvin to modify oxidative stress in human RPE cells was investigated by Koskela et al. [77]; by first evaluating the range of PS toxicity by exposing ARPE-19 cells to PS doses of 0.1–200 μM for 24 h, followed by a cell survival test. The ARPE-19 cells were then preincubated in pinosylvin for 24 h before being exposed to hydroquinone (HQ) without pinosylvin for another 24 h. Pinosylvin therapy at doses of 5 and 10 μM greatly improved cell survival against oxidative stress. Pinosylvin therapy elevated the production of HO-1, an enzyme with antioxidant, anti-inflammatory, and immunomodulatory abilities, which is positively associated with cell survival. Pinosylvin treatment did not influence the expression of Nrf2 or its target genes, p62 or GSTP1, while having a strong effect on the expression of HO-1, another Nrf2-controlled gene. RNA interference investigation verified the importance of Nrf2 and HO-1 in pinosylvin-mediated oxidative stress protection, whereas the contribution of p62 seemed minor at the gene expression and cell viability levels. The findings show that pinosylvin therapy protects against oxidative stress by inducing HO-1 in human RPE cells.

Pinosylvin is known to have an anti-inflammatory effect on endothelial cells. Hence, Kwon et al. [82] attempted to understand the exact process in their research. Pinosylvin was tested to determine if it increased COX or lipoxygenase (LOX) activity in THP-1 and U937 cells. Pinosylvin significantly increased LOX activity without affecting COX activity. Furthermore, it increased ALOX15 mRNA and protein levels, demonstrating that pinosylvin-induced LOX activity is due to increased ALOX15 expression. Pinosylvin appeared to enhance ERK and JNK phosphorylation in this cell signaling investigation. ERK and JNK inhibitors were observed to reduce ALOX15 expression and LPS-induced apoptosis produced by pinosylvin. Finally, pinosylvin promoted apoptosis in LPS-preconditioned leukocytes by increasing ALOX15 expression via ERK and JNK.

In cancer patients, metastases are a major cause of mortality [5]. Previous research revealed that pinosylvin has a potential cancer chemopreventive effect and suppresses the development of many human cancer cell lines by regulating cell cycle progression. In this study, the authors investigated the possible antimetastatic action of pinosylvin using in vitro and in vivo models. In cultured human fibrosarcoma HT1080 cells, pinosylvin inhibited the production of MMP-2, MMP-9, and membrane type 1-MMP. Pinosylvin has also been reported to interfere with HT1080 cell migration in colony dispersal and wound healing methods. Pinosylvin (10 mg/kg b.w., intraperitoneal treatment) effectively reduced tumor nodule growth and tumor weight in lung tissues in an in vivo model of spontaneous lung metastasis following injection of CT26 colon carcinoma into BALB/c mice. The study of tumors in lung tissue revealed that the antimetastatic impact of pinosylvin was associated with a decrease in the production of MMP-9 and COX-2 and the activation of ERK1/2 and Akt. These findings show that pinosylvin, via modulating MMPs, might be an effective inhibitor of tumor cell metastasis.

Park et al. [21] investigated the antiproliferative action of pinosylvin in human colorectal HCT-116 cancer cells to identify the underlying molecular processes. Pinosylvin inhibited HCT-116 cell proliferation by preventing the cell cycle from progressing from the G_1_ to the S phase, as well as downregulating cyclin D1, cyclin E, cyclin A, cyclin-dependent kinase 2 (CDK2), CDK4, c-Myc, and retinoblastoma protein (pRb) and the upregulation of p21^WAF1/CIP1^ and p53. Pinosylvin has also been shown to inhibit the activation of proteins involved in focal adhesion kinase and the phosphoinositide 3-kinase signaling system.

Pinosylvin, at high concentrations (100 μmol/L), was previously reported to promote cell death in bovine aortic endothelial cells. In the investigation conducted by Park et al. [22], it was attempted to reveal the role of pinosylvin in apoptosis, autophagy, and necrosis. Pinosylvin enhanced caspase-3 activation, nuclear condensation, and the “flip-flop” of phosphatidylserine at high concentrations, suggesting that pinosylvin triggers apoptosis. On the other hand, pinosylvin was found to suppress necrosis, a post-apoptotic process, based on flow cytometry data acquired using double-staining with annexin V and propidium iodide. Pinosylvin promoted LC3 conversion from LC3-I to LC3-II and p62 degradation, both of which are essential indications of autophagy. Furthermore, pinosylvin appeared to stimulate AMP-activated protein kinase (AMPK), and an AMPK inhibitor significantly reduced LC3 conversion. Pinosylvin reversed the inhibitory impact of an AMPK inhibitor. These findings imply that pinosylvin causes autophagy by activating AMPK. Additionally, an autophagy inhibitor was shown to enhance necrosis, which was later restored with pinosylvin, but the caspase-3 inhibitor had no impact on necrosis. These results show that pinosylvin-induced autophagy inhibits necrotic progression in endothelial cells.

In the study performed by Simard et al. [89], methanol extracts of *Pinus resinosa* wood containing pinosylvin were selectively cytotoxic against human lung cancer cells, A549 (IC_50_ = 41.6 μg/mL) and human colorectal adenocarcinoma cells, DLD-1 (IC_50_ = 47.4 μg/mL) compared to healthy cells, WS1 (IC_50_ = 130.11 μg/mL). Five known compounds were isolated and identified as: pinosylvin monomethyl ether (1), pinosylvin (2), pinosylvin dimethyl ether (3), pinobanksin (4), and (-)-norachelogenin using ^1^H-, ^13^C-NMR spectroscopy and HR-ESI-MS mass spectrometry (5). Compounds 1–5 were tested for their cytotoxicity against A549, DLD-1, and WS1. Compound 1 (pinosylvin monomethyl ether) had the highest cytotoxicity against both tumor and healthy cell lines, with IC_50_ values of 25.4, 20.1, and 34.3 μM for A549, DLD-1, and WS1, respectively.

Skinnider and Stoessl [88] investigated the effects of phytoalexins lubimin, (-)-maackiain, pinosylvin, and related chemicals dehydroloroglossol and hordatine M on the development of the human lymphoblastoid cell lines Molt and Raji. The authors found that (-)-maackiain, pinosylvin, and dehydroloroglossol all significantly inhibited cell proliferation. The inhibition of [3H] thymidine and [3H] leucine absorption in pinosylvin and dehydroloroglossol was studied and shown to be effective. Phytoalexins and similar chemicals are abundant in plants and may serve as a source of antineoplastic drugs.

Several tests were carried out in the Song et al. [87] investigation to determine how high concentrations of pinosylvin (50 μM) promotes endothelial cell death. Pinosylvin, at high concentrations, was demonstrated to promote endothelial cell death by increasing caspase-3 activity, phosphatidylserine flip-flop, and nuclear fragmentation. They discovered that high concentrations of pinosylvin increased caspase-3 activity, which was amplified by serum deprivation or treatment with 100 μM etoposide. They also found that high concentrations of pinosylvin stimulated the activation of c-Jun N-terminal kinase (JNK) and endothelial nitric oxide synthase (eNOS). They then conducted a series of tests to determine which signaling molecule was important in pinosylvin-induced apoptosis. Finally, they found that SP-600125, a JNK inhibitor, inhibited pinosylvin-induced endothelial cell death, whereas L-NAME, an eNOS inhibitor, had no impact. These findings suggest that JNK is implicated in pinosylvin-induced apoptosis. At high concentrations, pinosylvin promotes cell death through JNK activation.

Resveratrol (pinosylvin analogue) has been shown to promote cell death in leukemia cells at high doses (50–100 μmol/L). Song et al. [86] foudn that cell death was significantly increased from 50 to 100 μmol/L pinosylvin in THP1 and U937 cells. Pinosylvin also induced caspase-3 activation, phosphatidylserine flipflop, LC3II accumulation, LC3 puncta, and p62 degradation in THP1 and U937 cells. These findings suggest that pinosylvin-induced cell death may occur through apoptosis and autophagy. Furthermore, we discovered that pinosylvin inhibits AMP-activated protein kinase 1 (AMPK1) in leukemia cells. As a result, a link was found between AMPK1 downregulation and leukemic cell death. Inhibition of AMPK1 reduces pinosylvin-induced apoptosis and autophagy in leukemia cells, indicating that AMPK is a crucial regulator of leukemia cell death. Moreover, when AMPK1-overexpressed leukemia cells were compared to vector-transfected cells, the progression of autophagy and apoptosis were inhibited by pinosylvin. Overexpression of AMPK1 increased cell death, but caspase-3 inhibitors or autophagy inhibitors significantly reduced pinosylvin-induced cell death. These findings imply that reducing AMPK1 by pinosylvin increases cell death by apoptosis and autophagy in leukemic cells.

### 5.5. Neuroprotective Activity

Based on the fact that neuroprotection is a typical technique to reduce the damage of cerebral ischemia, Xu et al. [67] set out to assess the neuroprotective efficacy of pinosylvin. Pinosylvin therapy reduced cell death in OGD/R-damaged PC12 cells and enhanced brain function in MCAO/R rats. Pinosylvin decreased the number of depolarized cells (low mitochondrial membrane potential) in OGD/R-damaged PC12 cells, implying a role in improving mitochondrial function. Further research revealed that pinosylvin triggers PINK1/Parkin-mediated protective mitophagy and activates the Nrf2 pathway, as shown by increased protein levels of LC3 II, Beclin1, PINK1, and Parkin, as well as Nrf2 translocation to the nucleus. Pinosylvin provided neuroprotection by triggering PINK1/Parkin-mediated mitophagy to eliminate damaged mitochondria and by activating the Nrf2 pathway to attenuate oxidative stress-induced mitochondrial dysfunction.

### 5.6. Anti-Allergic Activity

An extract of the branches of *H. dulcis* (containing pinosylvin) was tested for its anti-allergic potential using the rat basophilic leukemia (RBL)-2H3 cell line and the passive cutaneous anaphylaxis (PCA) mouse model using various assays [45]. The extract inhibited hexosaminidase secretion (indicating degranulation) and histamine release in antigen-stimulated RBL-2H3 cells, with decreased expression and production of the inflammatory mediators COX-2 and PGE_2_, as well as the cytokines IL-4 and TNF-α, and suppression of NF-κB activation indicating the potential of the extract as a strong antiallergic agent.

## 6. Conclusions and Perspectives

Here, the main pharmacological characteristics and sources of pinosylvin have been documented and highlighted. Numerous published research has demonstrated that this natural molecule has exceptional biological properties, especially against tumor cell lines. Both molecular and cellular analyses revealed that pinosylvin blocks and inhibits the key pathways in nasopharyngeal cancer, prostate cancer, fibrosarcoma, colorectal cancer, and oral cancer with different target sites. This indicates that it may be a valuable anti-cancer drug component. Additionally, this molecule’s antimicrobial, anti-inflammatory, antioxidant, and anti-allergic properties may qualify it as an effective bioactive ingredient in the treatment of cancer and neurodegenerative diseases. Nevertheless, a deeper insight into its pharmacokinetics and pharmacodynamics is required for its introduction as a chemotherapy drug. Furthermore, its safety requires validation by further toxicological studies.

## Figures and Tables

**Table 1:** Sources of Pinosylvin.

Species	Extract/Essential Oil	References
*Pinus sylvestris*	Extract	[13,24,25,26,27,28,29,30,31,32,33]
*Pinus resinosa*	Extract	[9,28,31,34]
*Pinus banksiana*	Extract	[26,28,31]
*Pinus nigra* Arn.	Extract	[25,30]
*Pinus densiflora*	Extract	[35,36]
*Pinus sibirica*	Extract	[37]
*Pinus contorta*	Extract	[37]
*Pinus strobus*	Extract	[38,39,40]
*Pinus taeda*	Extract	[41]
*Pinus cembra*	Extract	[37]
*Pinus pinaster*	Extract	[42,43,44]
*Hovenia dulcis* Thunb.	Extract	[45]
*Picea glauca*	Extract	[9]
*Nothofagus* (Southern beeches)	Extract	[46,47]
*Stemona* cf. *pierrei*	Extract	[48]
*Arachis hypogaea*	Extract	[49]

**Table 2:** Anti-inflammatory effects of pinosylvin.

Experimental Approaches	Key Results	References
Western blot analysis and reverse transcription-polymerase chain reaction (RT-PCR)	Inhibited COX-2, iNOS protein and gene expression	[76]
Murine adipocytes model, cytotoxicity assays, lipid staining, western blotting, and ELISA assays	Attenuated adipogenesis and inflammation through downregulation of the expression of PPARγ, C/EBP and TNF-a-stimulated IL-6 secretion	[14]
Cell viability and RNA interference analysis	Protected (10 µM) cell survival from oxidative damage by promoting HO-1 induction	[77]
LPS-induced mouse macrophage RAW 264.7 cells	Suppressed COX-2-mediated PGE_2_ production (IC_50_ = 10.6 µM)	[78]
LPS-stimulated Macrophage cells Western blot analysis RT-PCR analysis	Inhibited LPS-induced iNOS protein and mRNA expression in dose-dependent manner (IC_50_ = 39.9 μM) Decreased the expression levels of interferon regulatory factor 3 (IRF-3) and interferon-E (IFN-E)	[79]
AITC-induced acute paw inflammation in mice model Fluo-3-AM assay and patch clamping	Reduced paw inflammation formation by inhibiting and attenuating IL-6 production at the site of inflammation	[15]
Adjuvant-induced arthritis in rats	Pinosylvin + MTX reduced oxidative stress by upregulating HO-1 expression in lungs and reducing plasma activity of thiobarbituricacid reactive substances (TBARS) and lipoxygenase (LOX) in the lungs	[16]
Primary cultures of human OA chondrocytes	Increased aggrecan expression Inhibited IL-6 production by attenuating NF-κB activity	[13]
AA in rats Chemiluminescence (CL) of the joint and myeloperoxidase (MPO) activity	Decreased HPV Reduced CL of the joint and MPO activity of the joint homogenate	[80]
Carrageenan-induced paw edema in male C57BL/6 mice	Reduced inflammatory response by downregulating the production of inflammatory cytokines IL6, MCP1, and NO	[11]
AA was induced in Lewis rats Fresh human blood neutrophils as model	Reduced the formation of oxidants, both extra- and intra-cellular Suppressed PKC activation induced by phorbol myristate acetate Reduced neutrophil countDecreased the amount of ROS (in vivo)	[81]
LPS-triggered apoptosis in the leukocyte	Enhanced apoptosis of LPS-preconditioned leukocytes via decreasing ALOX 15 expression mediated by ERK and JNK pathways	[82]
Humane monocytic THP-1 cell lines Western blotting analysis	Suppressed proinflammatory enzymes TNF-α and IL-8 by the inhibition of NF-κB activation	[66]
Murine and U937 Human macrophages model qRT-PCR and ELISA	Changed macrophage polarization from the proinflammatory M1 phenotype to the M2 phenotype Promoted resolution of inflammation and repair Enhanced PPAR-γ expression in IL-4 treated macrophages	[12]
LPS-induced mouse macrophage RAW 264.7 cells	Decreased inflammation on LPS-stimulated macrophages Inhibited PPARγ activity in vitro	[83]
Antigen-stimulated mast cell-like cell line rat basophilic leukemia (RBL)-2H3 and a passive cutaneous anaphylaxis (PCA) mouse model Degranulation assay RT-PCR, PCA Western blot analyses	Suppressed the release and expression of allergic and proinflammatory key enzymes (IL-4, TNF-α and PGE_2_, COX-2, NFKB1, and NFKB2) in a dose-dependent manner	[45]

**Table 3:** Pinosylvin antioxidant activity.

Origins	Cell Lines	Methods	Key Findings	References
Synthesized	Mouse model of oligoasthenospermia	Epididymal sperm concentration and motility evaluation Hormone level assessment Real-time PCR Western blot analysis Evaluation of the testicular levels of ROS and MDA	Decreased oxidative stress through glutathione peroxidase 3 drastically reduced oxidative stress (in vivo) by inhibiting the nuclear factor erythroid 2-related factor 2 (Nrf2)/antioxidant response element (ARE) pathway	[19]
Purchased	WT and NFE2L2 KO(NFE2L2^−/−^) mice strain	ERG recording and processing of signals OCT imaging Antioxidant capacity analysis Immunohistochemical staining Confocal imaging	Retained retinal function Decreased accumulation of ubiquitin-tagged proteins Decreased chronic oxidative stress Preserved retinal function and morphology in the NFE2L2 KO disease model Reduced the risk of age-related macular degeneration (AMD) and halted its development	[85]
Purchased	In vitro non-enzymatic assays	ORAC-FL assay ABTS assay FRAP assay	Strong antioxidant and free radical scavenging properties	[18]
Synthetized	AA model induced in Lewis rats	Oral administration of pinosylvin to AA induced animals Monitoring of the hind paw volume Monitoring of the luminol-enhanced chemiluminescence (CL) of the joint Monitoring of myeloperoxidase (MPO) activity in hind paw joint	Reduced HPV (at days 14 and 28) Reduced joint CL and MPO activity in joint homogenate	[80]
Not reported	Human retinal pigment epithelial cells (ARPE-19)	Toxicity assessment Oxidative stress assessment MTT assay Real-time PCR Nrf2 and p62 RNA interference	Improved cell viability against oxidative stress (5 and 10 µM) Validated the importance of Nrf2 and HO-1 in pinosylvin-mediated protection against oxidative stress	[77]
Synthesized	Bovine aortic endothelial cells (BAECs)	Measurement of apoptosis Measurement of caspase-3 activity Cell proliferation Western blot analysis Cell migrationAdhesion of THP-1 to BAECs	Activated endothelial nitric oxide synthase Impacted cell proliferation in endothelial cells Stimulated cell migration and tube formation Avoided inflammatory cardiovascular disorders	[1]
Synthesized	AA model induced in rats	Formation of reactive oxygen species Western blot analysis Measurement of ATP liberation Flow cytometry Effects of pinosylvin on arthritis	Reduced both extracellular and intracellular oxidant generation in isolated human neutrophils Inhibited PKC activation triggered by phorbol myristate acetate Increased the number of neutrophils in the blood of arthritic rats Improved whole blood chemiluminescence (both spontaneous and PMA-stimulated) Reduced the number of neutrophils and the number of reactive oxygen species in the blood	[81]
Not reported	AA model induced in rats	28 days of oral administration Changes in hind paw volume and arthrogram evaluation γ-glutamyltransferase (GGT) activity assessment Measurement of thiobarbituric acid reactive substances (TBARS)	Decreased the activity of GGT in the spleen Reduced the activity of GGT in joint tissue Exhibited moderate efficacy in preventing oxidative damage	[17]
Synthetized	AA model induced in rats	Assessment of hind paw volume Measurement of the C-reactive protein Monocyte chemotactic protein-1 (MCP-1) level measurement Plasma levels of thiobarbituric acid reactive substances (TBARS) and F2-isoprostanes measurement G-glutamyltransferase and lipoxygenase (LOX) activity evaluation	Increased NF-κB activation in the liver and lung, HO-1 expression and LOX activity in the lung, MCP-1 levels in plasma, and F2-isoprostane plasmatic levels Reduced the OS (an increase of HO-1 expression in the lung and reduction in plasmatic TBARS) Decreased the LOX activity in the lung	[16]

**Table 4:** Anticancer activities of pinosylvin.

Origins	Cell Lines	Methods	Key Findings	References
Not clear	THP1 and U937 monocytic cell lines	Trypan blue exclusion assay Cell sorting analysis RT-PCR Preparation of cell lysates Western blot analysis Detection of LC3 puncta DNA transfection	Increased (50–100 μmol/L) cell death Caused caspase-3 activation, phosphatidylserine flipping, LC3II accumulation, LC3 puncta, and p62 degradation Induced cell death Caused downregulation of AMP-activated protein kinase (AMPK) α1	[86]
Not reported	Bovine aortic endothelial cells	Apoptosis assay Western blot analysis Flow cytometry analysis Measurement of caspase-3 activity	Increased caspase-3 activity, phosphatidylserine flip-flop, and nuclear fragmentation Activated JNK and endothelial NO synthase	[87]
Synthesized	Molt and Raji lymphoblastoid cell lines.	Growth inhibitory action Cell count and viability DNA and protein synthesis assessment	Inhibited cell proliferation Inhibited [3H] thymidine and leucine uptake	[88]
*Pinus resinosa*	A549, DLD-1, and WS1 cells	Cytotoxicity assay	66 ± 10 < IC_50_ < 75 ± 14 μM	[89]
Synthesized	HCT 116 colorectal cancer cells	Proliferation inhibitory potential testing Cell cycle distribution analysis Western blot analysis RT-PCR Identification of gene expression cDNA microarray Electrophoretic mobility shift assay	Slowed cell growth Slowed cell cycle transition from G_1_ phase to S phase Decreased the levels of cyclin D1, cyclin E, CDK2, c-Myc, pRb, and p53 Stopped the activation of proteins involved in the FAK/c-Src/ERK signaling pathway and the PI3K/Akt/GSK-3b signaling pathway Inhibited b-nuclear catenin translocation	[21]
Synthesized	HT1080 human fibrosarcoma and Balb/c mice	RT-PCR Wound healing assay Colony dispersion assessement In vivo pulmonary metastasis method Gelatin zymography	Inhibited the production of matrix metalloproteinase (MMP)-2, MMP-9, and membrane type 1-MMP Reduced HT1080 cell migration Slowed tumor nodule growth and tumor weight in lung tissue Downregulated MMP-9 and cyclooxygenase-2 (COX-2) expression and ERK1/2 and Akt phosphorylation in lung carcinoma tissues	[5]
Not reported	ARPE-19 cells	Toxicity evaluation Oxidative stress assessment Cell viability RT-PCR Nrf2 and p62 RNA interference	Improved cell viability in the face of oxidative stress Increased HO-1 expression No effect on Nrf2 expression Protected against oxidative damage	[77]
Purchased	LNCaP-par and LNCaP-abl prostate cancer cells	High-throughput screening (HTS) Cell viability and apoptosis assays Gene expression analysisqPT-PCR	Inhibited androgen signaling and intracellular steroidogenesis in CRPC cells	[23]
Purchased	Nasal cavity cancer cells (RPMI 2650)	MTT assay Gap closure assay Cell migration assay Cell invasion assay Western blot analysis Proteome profiler human protease array	Suppressed migration and invasion of NPC039 and NPCBM cells at increasing doses Lowered the protein expression levels of MMP2 and MMP9 Decreased the enzyme activity of MMP2Reduced vimentin and N-cadherin expression (in NPC cells) Increased zonula occludens-1 and E-cadherin expression Inhibited NPC039 and NPCBM cell invasion and migration by modulating the p38, ERK1/2, and JNK1/2 pathways Inhibited NPC cell migration and invasion	[20]
Purchased	SCC-9 and HSC-3 cancer cells (tongue squamous)	MTT assay Wound closure assessment Gelatin zymography Cell migration and invasion evaluation Western blot analysis	Decreased the enzymatic activity of MMP-2 and lowered its protein level Raised the expression of TIMP-2 Stopped cancer cell growth in a wound-healing experiment Reduced ERK1/2 protein phosphorylation in SAS and SCC-9 cells	[20]
Not reported	Bovine aortic endothelial cells	Apoptosis experiment Western blot analysis Flow cytometry Measurement of caspase-3 activity Hoechst staining	Induced (100 μmol/L) cell death Boosted caspase-3 activation, nuclear condensation, and the “flip-flop” of phosphatidylserine (at high concentrations) Inhibited necrosis Promoted LC3 conversion from LC3-I to LC3-II and p62 degradation Stimulated AMP-activated protein kinase (AMPK) and an AMPK inhibitor Reversed the inhibitory impact of an AMPK inhibitor Induced autophagy via AMPK activation	[87]

## Data Availability

Not applicable.

## Abbreviations

AMD	Age-related macular degeneration
MAPK	Mitogen-activated protein kinase
JNK	Jun amino-terminal kinase
NF-κB	Nuclear factor kappa B
Nrf2	Nuclear erythroid 2-related factor 2
ARE	Antioxidant response element
HCT	Colorectal cancer cell
FAK	Focal adhesion kinase
ERK	Extracellular signal-regulated kinase
GSK3	Glycogen synthase kinase-3
PI3K/AKT	Phosphatidylinositol 3-kinase/Protein kinase B
MMP	Matrix metalloproteinase
CX	Cyclohexane
EtOAc	Ethyl acetate
DRIFT	Diffuse reflectance fourier transform infrared spectroscopy
NIR	Near-infrared spectroscopy
FID	Flame ionization detection
GC	Gas chromatography
GC-MS	Gas chromatography-mass spectrometry
MIC	Minimal inhibitory concentration
EtBr	Ethidium bromide
Eps	Efflux pumps
MRSA	Methicillin-resistant *Staphylococcus aureus*;
ATTC	American type culture collection;
PGE2	Prostaglandin E2
NO	Nitric oxide
COX	Cyclooxygenase
iNOS	Inducible nitric oxide synthase
IL6	Interleukin 6
MCP1	Monocyte chemotactic protein 1
LPS	Lipopolysaccharide
IRF-3	Interferon regulatory factor 3
IFN-E	Interferon-E
TRIF	TIR-domain-containing adapter-inducing interferon-β
HO-1	Heme oxygenase-1
MTX	Methotrexate
TBARS	Plasmatic thiobarbituric acid-reactive substances
LOX	Lipoxygenase
TRPA1	Ankyrin subtype 1 protein
AA	Adjuvant arthritis
GGT	Glutamyltransferase
CRP	C-Reactive protein
PKC	Protein kinase C
GSTP1	Glutathione S-transferase pi 1
qRT-PCR	Quantitative reverse transcription polymerase chain reaction
HPV	Hind paw volume
CL	Chemiluminescence
MPO	Myeloperoxidase
LH	Luteinizing hormone
FSH	Follicle-stimulating hormone
ELISA	Enzyme-linked immunosorbent assay
NFE2L2	Nuclear factor erythroid 2 like 2
TIMP-2	Tissue inhibitor of metalloproteinase-2
NPC	Nasopharyngeal carcinoma
CRPC	Castration-resistant prostate cancer
PSME	Pinosylvin methyl ether
HQ	Hydroquinone
CDK2	Cyclin-dependent kinase 2
pRb	Retinoblastoma protein
AMPK	AMP-activated protein kinase
eNOS	Endothelial nitric oxide synthase
RBL	Rat basophilic leukemia
PCA	Passive cutaneous anaphylaxis
LC3-II	Microtubule associated protein 1 light chain 3-II

## References

[1] Jeong E., Lee H.-R., Pyee J., Park H. (2013). Pinosylvin Induces Cell Survival, Migration and Anti-Adhesiveness of Endothelial Cells via Nitric Oxide Production: Pinosylvin is a vasoregulating compound. Phytother. Res..

[2] Riviere C., Pawlus A.D., Merillon J.-M. (2012). Natural Stilbenoids: Distribution in the Plant Kingdom and Chemotaxonomic Interest in Vitaceae. Nat. Prod. Rep..

[3] Castelli G., Bruno F., Vitale F., Roberti M., Colomba C., Giacomini E., Guidotti L., Cascio A., Tolomeo M. (2016). In Vitro Antileishmanial Activity of Trans-Stilbene and Terphenyl Compounds. Exp. Parasitol..

[4] Akinwumi B.C., Bordun K.-A.M., Anderson H.D. (2018). Biological Activities of Stilbenoids. Int. J. Mol. Sci..

[5] Park E.-J., Park H.J., Chung H.-J., Shin Y., Min H.-Y., Hong J.-Y., Kang Y.-J., Ahn Y.-H., Pyee J.-H., Kook Lee S. (2012). Antimetastatic Activity of Pinosylvin, a Natural Stilbenoid, Is Associated with the Suppression of Matrix Metalloproteinases. J. Nutr. Biochem..

[6] Plumed-Ferrer C., Väkeväinen K., Komulainen H., Rautiainen M., Smeds A., Raitanen J.-E., Eklund P., Willför S., Alakomi H.-L., Saarela M. (2013). The Antimicrobial Effects of Wood-Associated Polyphenols on Food Pathogens and Spoilage Organisms. Int. J. Food Microbiol..

[7] Silva F., Domingues F.C., Nerín C. (2018). Control Microbial Growth on Fresh Chicken Meat Using Pinosylvin Inclusion Complexes Based Packaging Absorbent Pads. LWT.

[8] Sousa V., Luís Â., Oleastro M., Domingues F., Ferreira S. (2019). Polyphenols as Resistance Modulators in Arcobacter Butzleri. Folia Microbiol..

[9] Celimene C.C., Micales J.A., Ferge L., Young R.A. (1999). Efficacy of Pinosylvins against White-Rot and Brown-Rot Fungi. Holzforschung.

[10] Lee D.G., Lee S.J., Rodriguez J.P., Kim I.H., Chang T., Lee S. (2017). Antifungal Activity of Pinosylvin from Pinus Densiflora on Turfgrass Fungal Diseases. J. Appl. Biol. Chem..

[11] Eräsalo H., Hämäläinen M., Leppänen T., Mäki-Opas I., Laavola M., Haavikko R., Yli-Kauhaluoma J., Moilanen E. (2018). Natural Stilbenoids Have Anti-Inflammatory Properties in Vivo and down-Regulate the Production of Inflammatory Mediators NO, IL6, and MCP1 Possibly in a PI3K/Akt-Dependent Manner. J. Nat. Prod..

[12] Kivimäki K., Leppänen T., Hämäläinen M., Vuolteenaho K., Moilanen E. (2021). Pinosylvin Shifts Macrophage Polarization to Support Resolution of Inflammation. Molecules.

[13] Laavola M., Nieminen R., Leppänen T., Eckerman C., Holmbom B., Moilanen E. (2015). Pinosylvin and Monomethylpinosylvin, Constituents of an Extract from the Knot of Pinus Sylvestris, Reduce Inflammatory Gene Expression and Inflammatory Responses in Vivo. J. Agric. Food Chem..

[14] Modi S., Yaluri N., Kokkola T. (2018). Strigolactone GR24 and Pinosylvin Attenuate Adipogenesis and Inflammation of White Adipocytes. Biochem. Biophys. Res. Commun..

[15] Moilanen L.J., Hämäläinen M., Lehtimäki L., Nieminen R.M., Muraki K., Moilanen E. (2016). Pinosylvin Inhibits TRPA 1-Induced Calcium Influx In Vitro and TRPA 1-Mediated Acute Paw Inflammation In Vivo. Basic Clin. Pharmacol. Toxicol..

[16] Bauerova K., Acquaviva A., Ponist S., Gardi C., Vecchio D., Drafi F., Arezzini B., Bezakova L., Kuncirova V., Mihalova D. (2015). Markers of Inflammation and Oxidative Stress Studied in Adjuvant-Induced Arthritis in the Rat on Systemic and Local Level Affected by Pinosylvin and Methotrexate and Their Combination. Autoimmunity.

[17] Drafi F., Bauerova K., Kuncirova V., Ponist S., Mihalova D., Fedorova T., Harmatha J., Nosal R. (2012). Pharmacological Influence on Processes of Adjuvant Arthritis: Effect of the Combination of an Antioxidant Active Substance with Methotrexate. Interdiscip. Toxicol..

[18] Rodríguez-Bonilla P., Gandía-Herrero F., Matencio A., García-Carmona F., López-Nicolás J.M. (2017). Comparative Study of the Antioxidant Capacity of Four Stilbenes Using ORAC, ABTS+, and FRAP Techniques. Food Anal. Methods.

[19] Wang C., Sang M., Gong S., Yang J., Cheng C.Y., Sun F. (2020). Two Resveratrol Analogs, Pinosylvin and 4,4′-Dihydroxystilbene, Improve Oligoasthenospermia in a Mouse Model by Attenuating Oxidative Stress via the Nrf2-ARE Pathway. Bioorg. Chem..

[20] Chen M.-K., Liu Y.-T., Lin J.-T., Lin C.-C., Chuang Y.-C., Lo Y.-S., Hsi Y.-T., Hsieh M.-J. (2019). Pinosylvin Reduced Migration and Invasion of Oral Cancer Carcinoma by Regulating Matrix Metalloproteinase-2 Expression and Extracellular Signal-Regulated Kinase Pathway. Biomed. Pharmacother..

[21] Park E.-J., Chung H.-J., Park H.J., Kim G.D., Ahn Y.-H., Lee S.K. (2013). Suppression of Src/ERK and GSK-3/β-Catenin Signaling by Pinosylvin Inhibits the Growth of Human Colorectal Cancer Cells. Food Chem. Toxicol..

[22] Park J., Pyee J., Park H. (2014). Pinosylvin at a High Concentration Induces AMPK-Mediated Autophagy for Preventing Necrosis in Bovine Aortic Endothelial Cells. Can. J. Physiol. Pharmacol..

[23] Ketola K., Viitala M., Kohonen P., Fey V., Culig Z., Kallioniemi O., Iljin K. (2016). High-Throughput Cell-Based Compound Screen Identifies Pinosylvin Methyl Ether and Tanshinone IIA as Inhibitors of Castration-Resistant Prostate Cancer. J. Mol. Biochem..

[24] Chambers V.H. (1945). British Bees and Wind-Borne Pollen. Nature.

[25] Ioannidis K., Melliou E., Alizoti P., Magiatis P. (2017). Identification of Black Pine (*Pinus nigra* Arn.) Heartwood as a Rich Source of Bioactive Stilbenes by QNMR. J. Sci. Food Agric..

[26] Rowe J.W., Bower C.L., Wagner E.R. (1969). Extractives of Jack Pine Bark: Occurrence of Cis- and Trans-Pinosylvin Dimethyl Ether and Ferulic Acid Esters. Phytochemistry.

[27] Seppänen S.K., Syrjälä L., Von Weissenberg K., Teeri T.H., Paajanen L., Pappinen A. (2004). Antifungal Activity of Stilbenes in in Vitro Bioassays and in Transgenic Populus Expressing a Gene Encoding Pinosylvin Synthase. Plant Cell Rep..

[28] Pietarinen S.P., Willför S.M., Ahotupa M.O., Hemming J.E., Holmbom B.R. (2006). Knotwood and Bark Extracts: Strong Antioxidants from Waste Materials. J. Wood Sci..

[29] Hovelstad H., Leirset I., Oyaas K., Fiksdahl A. (2006). Screening Analyses of Pinosylvin Stilbenes, Resin Acids and Lignans in Norwegian Conifers. Molecules.

[30] Vek V., Poljanšek I., Humar M., Willför S., Oven P. (2020). In Vitro Inhibition of Extractives from Knotwood of Scots Pine (*Pinus sylvestris*) and Black Pine (*Pinus nigra*) on Growth of *Schizophyllum commune*, *Trametes versicolor*, *Gloeophyllum trabeum* and *Fibroporia Vaillantii*. Wood Sci. Technol..

[31] Lindberg L.E., Willför S.M., Holmbom B.R. (2004). Antibacterial Effects of Knotwood Extractives on Paper Mill Bacteria. J. Ind. Microbiol. Biotechnol..

[32] Verkasalo E., Möttönen V., Roitto M., Vepsäläinen J., Kumar A., Ilvesniemi H., Siwale W., Julkunen-Tiitto R., Raatikainen O., Sikanen L. (2021). Extractives of Stemwood and Sawmill Residues of Scots Pine (*Pinus sylvestris* L.) for Biorefining in Four Climatic Regions in Finland-Phenolic and Resin Acid Compounds. Forests.

[33] Bergström B. (2003). Chemical and Structural Changes during Heartwood Formation in *Pinus sylvestris*. Forestry.

[34] François S., Jean L., Serge L., Vakhtang M., André P. (2008). Inhibition of Cholinesterase and Amyloid-&bgr; Aggregation by Resveratrol Oligomers from *Vitis amurensis*. Phytother. Res..

[35] Kodan A., Kuroda H., Sakai F. (2002). A Stilbene Synthase from Japanese Red Pine (*Pinus densiflora*): Implications for Phytoalexin Accumulation and down-Regulation of Flavonoid Biosynthesis. Proc. Natl. Acad. Sci. USA.

[36] Dumas M.T., Hubbes M., Strunz G.M. (1983). Identification of Some Compounds Associated with Resistance of *Pinus densiflora* to *Fomes annosus*. Eur. J. For. Pathol..

[37] Willför S.M., Ahotupa M.O., Hemming J.E., Reunanen M.H.T., Eklund P.C., Sjöholm R.E., Eckerman C.S.E., Pohjamo S.P., Holmbom B.R. (2003). Antioxidant Activity of Knotwood Extractives and Phenolic Compounds of Selected Tree Species. J. Agric. Food Chem..

[38] Raiber S., Schröder G., Schröder J. (1995). Molecular and Enzymatic Characterization of Two Stilbene Synthases from Eastern White Pine (*Pinus strobus*) A Single Arg/His Difference Determines the Activity and the PH Dependence of the Enzymes. FEBS Lett..

[39] Hwang H.S., Han J.Y., Choi Y.E. (2021). Enhanced Accumulation of Pinosylvin Stilbenes and Related Gene Expression in *Pinus Strobus* after Infection of Pine Wood Nematode. Tree Physiol..

[40] Koo H.B., Hwang H.S., Han J.Y., Cheong E.J., Kwon Y.S., Choi Y.E. (2022). Enhanced Production of Pinosylvin Stilbene with Aging of *Pinus strobus* Callus and Nematicidal Activity of Callus Extracts against Pinewood Nematodes. Sci. Rep..

[41] Hemingway R.W., Mcgraw G.W., Barras S.J. (1977). Polyphenols in Ceratocystis Minor Infected *Pinus taeda*: Fungal Metabolites, Phloem and Xylem Phenols. J. Agric. Food Chem..

[42] Gabaston J., Leborgne C., Waffo-Téguo P., Pedrot E., Richard T., Mérillon J.M., Valls Fonayet J. (2020). Separation and Isolation of Major Polyphenols from Maritime Pine (*Pinus pinaster*) Knots by Two-Step Centrifugal Partition Chromatography Monitored by LC-MS and NMR Spectroscopy. J. Sep. Sci..

[43] Gabaston J., Richard T., Cluzet S., Palos Pinto A., Dufour M.C., Corio-Costet M.F., Mérillon J.M. (2017). *Pinus pinaster* Knot: A Source of Polyphenols against *Plasmopara viticola*. J. Agric. Food Chem..

[44] Conde E., Fang W., Hemming J., Willför S., Domínguez H., Parajó J.C. (2014). Recovery of Bioactive Compounds from *Pinus pinaster* Wood by Consecutive Extraction Stages. Wood Sci. Technol..

[45] Lim S.J., Kim M., Randy A., Nho C.W. (2015). Inhibitory Effect of the Branches of *Hovenia dulcis* Thunb. and Its Constituent Pinosylvin on the Activities of IgE-Mediated Mast Cells and Passive Cutaneous Anaphylaxis in Mice. Food Funct..

[46] Wollenweber E., Stevens J.F., Dörr M., Rozefelds A.C. (2003). Taxonomic Significance of Flavonoid Variation in Temperate Species of Nothofagus. Phytochemistry.

[47] Gyeltshen T., Jordan G.J., Smith J.A., Bissember A.C. (2022). Natural Products Isolation Studies of the Paleoendemic Plant Species *Nothofagus gunnii* and *Nothofagus cunninghamii*. Fitoterapia.

[48] Kostecki K., Engelmeier D., Pacher T., Hofer O., Vajrodaya S., Greger H. (2004). Dihydrophenanthrenes and Other Antifungal Stilbenoids from *Stemona* cf. Pierrei. Phytochemistry.

[49] Schöppner A., Kindl H. (1984). Purification and Properties of a Stilbene Synthase from Induced Cell Suspension Cultures of Peanut. J. Biol. Chem..

[50] Poljanšek I., Oven P., Vek V., Raitanen J.E., Hemming J., Willför S. (2019). Isolation of Pure Pinosylvins from Industrial Knotwood Residue with Non-Chlorinated Solvents. Holzforschung.

[51] Jorgensen E., Balsillie D. (1969). Formation of Heartwood Phenols in Callus Tissue Cultures of Red Pine (*Pinus resinosa*). Can. J. Bot..

[52] Lange B.M., Trost M., Heller W., Langebartels C., Sandermann H. (1994). Elicitor-Induced Formation of Free and Cell-Wall-Bound Stilbenes in Cell-Suspension Cultures of Scots Pine (*Pinus sylvestris* L.). Planta.

[53] Makrides S.C. (1996). Strategies for Achieving High-Level Expression of Genes in *Escherichia coli*. Microbiol. Rev..

[54] Wu J., Liu P., Fan Y., Bao H., Du G., Zhou J., Chen J. (2013). Multivariate Modular Metabolic Engineering of *Escherichia coli* to Produce Resveratrol from L-Tyrosine. J. Biotechnol..

[55] Watts K.T., Lee P.C., Schmidt-Dannert C. (2006). Biosynthesis of Plant-Specific Stilbene Polyketides in Metabolically Engineered *Escherichia coli*. BMC Biotechnol..

[56] Van Summeren-Wesenhagen P.V., Marienhagen J. (2015). Metabolic Engineering of *Escherichia coli* for the Synthesis of the Plant Polyphenol Pinosylvin. Appl. Environ. Microbiol..

[57] Xu J.Y., Xu Y., Chu X., Tan M., Ye B.C. (2018). Protein Acylation Affects the Artificial Biosynthetic Pathway for Pinosylvin Production in Engineered *E. coli*. ACS Chem. Biol..

[58] Liang J.-L., Guo L.-Q., Lin J.-F., He Z.-Q., Cai F.-J., Chen J.-F. (2016). A Novel Process for Obtaining Pinosylvin Using Combinatorial Bioengineering in *Escherichia coli*. World J. Microbiol. Biotechnol..

[59] Salas-Navarrete C., Hernández-Chávez G., Flores N., Martínez L.M., Martinez A., Bolívar F., Barona-Gomez F., Gosset G. (2018). Increasing Pinosylvin Production in *Escherichia coli* by Reducing the Expression Level of the Gene FabI-Encoded Enoyl-Acyl Carrier Protein Reductase. Electron. J. Biotechnol..

[60] Katsuyama Y., Funa N., Horinouchi S. (2007). Precursor-Directed Biosynthesis of Stilbene Methyl Ethers in *Escherichia coli*. Biotechnol. J..

[61] Wang S., Zhang S., Xiao A., Rasmussen M., Skidmore C., Zhan J. (2015). Metabolic Engineering of *Escherichia coli* for the Biosynthesis of Various Phenylpropanoid Derivatives. Metab. Eng..

[62] Holmgren A., Bergström B., Gref R., Ericsson A. (1999). Detection of Pinosylvins in Solid Wood of Scots Pine Using Fourier Transform Raman and Infrared Spectroscopy. J. Wood Chem. Technol..

[63] Roupe K., Halls S., Davies N.M. (2005). Determination and Assay Validation of Pinosylvin in Rat Serum: Application to Drug Metabolism and Pharmacokinetics. J. Pharm. Biomed. Anal..

[64] Ekeberg D., Flæte P.O., Eikenes M., Fongen M., Naess-Andresen C.F. (2006). Qualitative and Quantitative Determination of Extractives in Heartwood of Scots Pine (*Pinus sylvestris* L.) by Gas Chromatography. J. Chromatogr. A.

[65] Preusz M., Tříska J., Vrchotová N., Vilímek J., Enei F., Preusz K. (2019). Chemical Profile of Organic Residues from Ancient Amphoras Found in Pyrgi and Castrum Novum, Tyrrhenian Sea (Italy). J. Archaeol. Sci. Rep..

[66] Lee S.K., Lee H.J., Min H.Y., Park E.J., Lee K.M., Ahn Y.H., Cho Y.J., Pyee J.H. (2005). Antibacterial and Antifungal Activity of Pinosylvin, a Constituent of Pine. Fitoterapia.

[67] Xu H., Deng R., Li E.T.S., Shen J., Wang M. (2020). Pinosylvin Provides Neuroprotection against Cerebral Ischemia and Reperfusion Injury through Enhancing PINK1/Parkin Mediated Mitophagy and Nrf2 Pathway. J. Funct. Foods.

[68] Sharifi-Rad J., Dey A., Koirala N., Shaheen S., El Omari N., Salehi B., Goloshvili T., Silva N.C.C., Bouyahya A., Vitalini S. (2021). *Cinnamomum* Species: Bridging Phytochemistry Knowledge, Pharmacological Properties and Toxicological Safety for Health Benefits. Front. Pharmacol..

[69] Bouyahya A., Chamkhi I., Benali T., Guaouguaou F.-E., Balahbib A., El Omari N., Taha D., Belmehdi O., Ghokhan Z., El Menyiy N. (2021). Traditional Use, Phytochemistry, Toxicology, and Pharmacology of *Origanum majorana* L.. J. Ethnopharmacol..

[70] Bouyahya A., El Omari N., Elmenyiy N., Guaouguaou F.-E., Balahbib A., El-Shazly M., Chamkhi I. (2020). Ethnomedicinal Use, Phytochemistry, Pharmacology, and Toxicology of *Ajuga iva* (L.) Schreb. J. Ethnopharmacol..

[71] Marmouzi I., Bouyahya A., Ezzat S.M., El Jemli M., Kharbach M. (2021). The Food Plant *Silybum marianum* (L.) Gaertn.: Phytochemistry, Ethnopharmacology and Clinical Evidence. J. Ethnopharmacol..

[72] De Bruijn W.J., Araya-Cloutier C., Bijlsma J., de Swart A., Sanders M.G., de Waard P., Gruppen H., Vincken J.-P. (2018). Antibacterial Prenylated Stilbenoids from Peanut (*Arachis hypogaea*). Phytochem. Lett..

[73] Silva F., Nerín C., Domingues F.C. (2015). Stilbene Phytoallexins Inclusion Complexes: A Natural-Based Strategy to Control Foodborne Pathogen Campylobacter. Food Control.

[74] Bouyahya A., Omari N.E., El Hachlafi N., Jemly M.E., Hakkour M., Balahbib A., El Menyiy N., Bakrim S., Naceiri Mrabti H., Khouchlaa A. (2022). Chemical Compounds of Berry-Derived Polyphenols and Their Effects on Gut Microbiota, Inflammation, and Cancer. Molecules.

[75] Bouyahya A., Guaouguaou F.-E., El Omari N., El Menyiy N., Balahbib A., El-Shazly M., Bakri Y. (2021). Anti-Inflammatory and Analgesic Properties of Moroccan Medicinal Plants: Phytochemistry, in Vitro and in Vivo Investigations, Mechanism Insights, Clinical Evidences and Perspectives. J. Pharm. Anal..

[76] Park J.-H., Choi G.J., Jang K.S., Lim H.K., Kim H.T., Cho K.Y., Kim J.-C. (2005). Antifungal Activity against Plant Pathogenic Fungi of Chaetoviridins Isolated from *Chaetomium globosum*. FEMS Microbiol. Lett..

[77] Koskela A., Reinisalo M., Hyttinen J.M., Kaarniranta K., Karjalainen R.O. (2014). Pinosylvin-Mediated Protection against Oxidative Stress in Human Retinal Pigment Epithelial Cells. Mol. Vis..

[78] Park E.-J., Min H.-Y., Ahn Y.-H., Bae C.-M., Pyee J.-H., Lee S.K. (2004). Synthesis and Inhibitory Effects of Pinosylvin Derivatives on Prostaglandin E2 Production in Lipopolysaccharide-Induced Mouse Macrophage Cells. Bioorg. Med. Chem. Lett..

[79] Park E.-J., Min H.-Y., Chung H.-J., Ahn Y.-H., Pyee J.-H., Lee S.K. (2011). Pinosylvin Suppresses LPS-Stimulated Inducible Nitric Oxide Synthase Expression via the MyD88-Independent, but TRIF-Dependent Downregulation of IRF-3 Signaling Pathway in Mouse Macrophage Cells. Cell. Physiol. Biochem..

[80] Mačičková T., Drábiková K., Nosal R., Bauerová K., Mihálová D., Harmatha J., Pečivová J. (2010). In Vivo Effect of Pinosylvin and Pterostilbene in the Animal Model of Adjuvant Arthritis. Neuroendocrinol. Lett..

[81] Jančinová V., Perečko T., Nosáľ R., Harmatha J., Šmidrkal J., Drábiková K. (2012). The Natural Stilbenoid Pinosylvin and Activated Neutrophils: Effects on Oxidative Burst, Protein Kinase C, Apoptosis and Efficiency in Adjuvant Arthritis. Acta Pharmacol. Sin..

[82] Kwon O., Seo Y., Park H. (2018). Pinosylvin Exacerbates LPS-Induced Apoptosis via ALOX 15 Upregulation in Leukocytes. BMB Rep..

[83] Schuster R., Holzer W., Doerfler H., Weckwerth W., Viernstein H., Okonogi S., Mueller M. (2016). Cajanus Cajan–a Source of PPARγ Activators Leading to Anti-Inflammatory and Cytotoxic Effects. Food Funct..

[84] Park E.-J., Ahn Y.-H., Pyee J.-H., Park H.J., Chung H.-J., Min H.-Y., Hong J.-Y., Kang Y.-J., Bae I.-K., Lee S.K. (2005). Suppressive Effects of Pinosylvin, a Natural Stilbenoid, on Cyclooxygenase-2 and Inducible Nitric Oxide Synthase and the Growth Inhibition of Cancer Cells. Cancer Res..

[85] Tamminen T., Koskela A., Toropainen E., Gurubaran I.S., Winiarczyk M., Liukkonen M., Paterno J.J., Lackman P., Sadeghi A., Viiri J. (2021). Pinosylvin Extract Retinari™ Sustains Electrophysiological Function, Prevents Thinning of Retina, and Enhances Cellular Response to Oxidative Stress in NFE2L2 Knockout Mice. Oxid. Med. Cell. Longev..

[86] Song J., Seo Y., Park H. (2018). Pinosylvin Enhances Leukemia Cell Death via Down-Regulation of AMPKα Expression: Anti-Cancer Activity of Pinosylvin. Phytother. Res..

[87] Jina S., Jinsun P., Eunsil J., A-Young S., Jaeho P., Heonyong P. (2015). Apoptotic Effect of Pinosylvin at a High Concentration Regulated by C-Jun N-Terminal Kinase in Bovine Aortic Endothelial Cells. J. Life Sci..

[88] Skinnider L., Stoessl A. (1986). The Effect of the Phytoalexins, Lubimin, (−)-Maackiain, Pinosylvin, and the Related Compounds Dehydroloroglossol and Hordatine M on Human Lymphoblastoid Cell Lines. Experientia.

[89] Simard F., Legault J., Lavoie S., Mshvildadze V., Pichette A. (2008). Isolation and Identification of Cytotoxic Compounds from the Wood of *Pinus resinosa*. Phytother. Res..

[90] Chuang Y.-C., Hsieh M.-C., Lin C.-C., Lo Y.-S., Ho H.-Y., Hsieh M.-J., Lin J.-T. (2021). Pinosylvin Inhibits Migration and Invasion of Nasopharyngeal Carcinoma Cancer Cells via Regulation of Epithelial-mesenchymal Transition and Inhibition of MMP-2. Oncol. Rep..

